# A Technique to Augment Arthroscopic Bankart Repair With or Without a Metal Block: A Comparison

**DOI:** 10.3390/jcm14020616

**Published:** 2025-01-18

**Authors:** Paul Vedrenne, Mohamad K. Moussa, Kévin Picard, Thomas Bauer, Alexandre Hardy

**Affiliations:** 1 CHU Henri Mondor, 94000 Creteil, France; 2 Clinique du Sport, 75005 Paris, France; 3 Hôpital Saint Antoine, 75012 Paris, France; 4 Hôpital Ambroise Paré, 92100 Boulogne Billancourt, France; thomas.bauer@aphp.fr

**Keywords:** implants, experimental, joint instability, arthroscopy, Bankart lesions, shoulder joint

## Abstract

**Introduction:** Arthroscopic Bankart repair (ABR) is associated with an increased failure rate over time. The Recenter implant, a metal block, is designed to reinforce capsulolabral repair. The aim of this study was to evaluate whether the addition of the Recenter implant to ABR reduces the rate of recurrence in patients with glenohumeral anterior instability. **Materials and Methods:** This was a retrospective, multicentric case–control study focusing on patients surgically treated for anterior shoulder instability from February 2012 to November 2019. This study compared patients undergoing ABR augmented with the “Recenter” implant (augmented ABR group) against those receiving traditional ABR. Primary outcomes measured included recurrence rates. Secondary outcome measures included functional scores (Walch–Duplay and the subjective shoulder test [SST], the auto Rowe score, satisfaction, pain, and the presence or absence of subjective subluxation and apprehension), return to sports, the range of motion, as well as other complications. **Results:** Thirty-two patients with augmented ABR were compared to forty-eight patients in the traditional ABR group, with mean follow-up periods of 5.2 ± 1.3 years and 6.1 ± 1.5 years, respectively. Three patients (9.4%) experienced recurrence in the “Recenter” group, versus eight (16.7%) in the other group (*p* > 0.05). The Walch–Duplay score was 70.2 ± 8.2 in the “Recenter” group and 64.2 ± 8 in the control group (*p* > 0.05). The SST score out of 100 was, respectively, 84.6 ± 6 and 81.5 ± 5.5 (*p* = 0.05). There were no early complications in the implant group. No statistically significant differences were observed between the two groups for the other outcomes. **Conclusions:** ABR safely restores shoulder stability in selected patients with subcritical glenoid bone deficiency. However, the addition of the Recenter metal implant did not improve outcomes compared to traditional Bankart repair and introduced presumed significant surgical time, technical challenges, and additional costs.

## 1. Introduction

Arthroscopic Bankart repair (ABR) is the most used surgical technique for the management of anterior shoulder instability [1,2]. Provided careful patient selection, this procedure has been reported to lead to satisfactory outcomes with low rates of early complications [3]. However, the effectiveness of this procedure has been shown to decrease over time and may lead to late recurrent instability [4,5].

Griffith et al. reported that recurrent anterior shoulder instability is associated with a bony defect in more than 80% of cases [6]. The treatment of significant bone loss is commonly treated using a coracoid, iliac crest or distal clavicle bone autograft. Although these procedures have been proven to provide excellent results in terms of recurrent instability [7], they are known to be associated with an increased complication rate, including infection, graft non-union, graft lysis, and degenerative joint disease [1,8,9]. Even subcritical bone loss, for example, small glenoid lesions (<20%), lead to increased anterior humeral translation, higher glenoid contact pressures, and increased recurrent instability [10,11,12]. The assessment of small bony defects remains unclear and may lead to the overuse of the Latarjet procedure in some cases [13].

Moroder et al. [14] described a Bankart-Plus procedure which added an allogeneic demineralized spongy bone matrix between the glenoid neck and the labrum to compensate for the glenoid bone loss by increasing backside support of the labrum and thus its stabilizing effect; however, no clinical results have been reported to date. Iizawa et al. [15] reported on the advantages of bone augmentation with ABR; however, this iliac crest autograft can be associated with residual donor site pain and the allograft can be both expensive and difficult to obtain. For subcritical glenoid bone loss (<20%), several other techniques have already been described to improve stability in addition to ABR, such as Remplissage [16], the long head of the biceps transfer to the glenoid (dynamic anterior stabilization) [17], and the autologous scapular spine bone graft [18].

**Figure 1:**
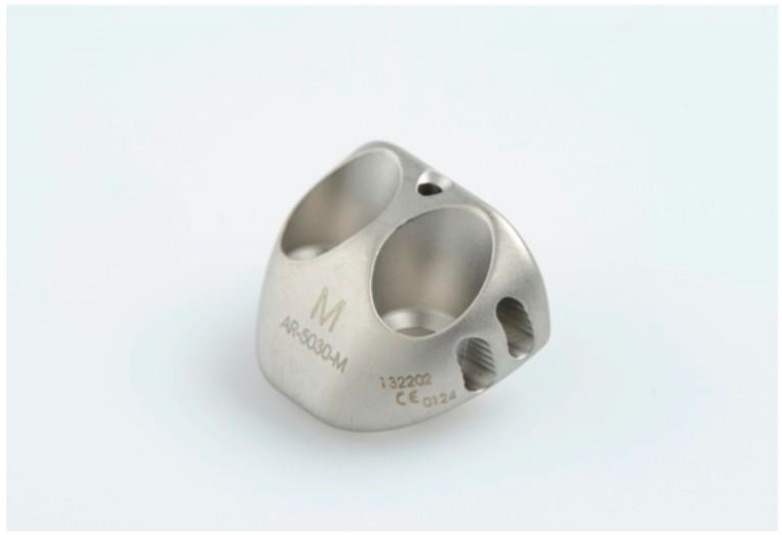
Prosthetic block: the Recenter.

The purpose of this study was to compare the clinical results of an ABR augmentation with a prosthetic block (Figure 1) called Recenter (Arthrex, Naples, FL, USA) with standard ABR. We hypothesized that the Recenter would result in better functional outcomes and a lower recurrent instability rate.

## 2. Materials and Methods

### 2.1. Study Design

This was a retrospective, multicentric, case–control study, conducted between February 2012 and November 2019, targeting consecutive patients treated surgically for anterior shoulder instability.

### 2.2. Ethical Consideration

Institutional review board approval was obtained for this study. Written informed consent to participate in the study was obtained from patients.

### 2.3. Inclusion and Exclusion Criteria

Inclusion criteria for the “Recenter” group included any patient who underwent augmented ABR at the participating centers during the inclusion period. Inclusion criteria for the control group were any patient who underwent ABR between February 2012 and March 2016 and between November 2017 and November 2019 in the main participating center. These time limits corresponded to the first available operative report in the center’s computer system and a minimum follow-up of two years.

Exclusion criteria included any revision surgery, Hill–Sachs lesion filling, indications other than for a form of anterior instability, and incomplete medical records.

### 2.4. Participants and Flowchart

A group of cases and a group of controls were retrospectively assembled from a pool of 101 consecutive patients who underwent surgery for glenohumeral instability and met the inclusion criteria.

Participants eligible for the “Recenter” group consisted of 38 patients who underwent augmented ABR with a Recenter device in four different centers between April 2016 and October 2017. Six patients were lost to follow-up leaving thirty-two patients included in this group.

**Figure 2:**
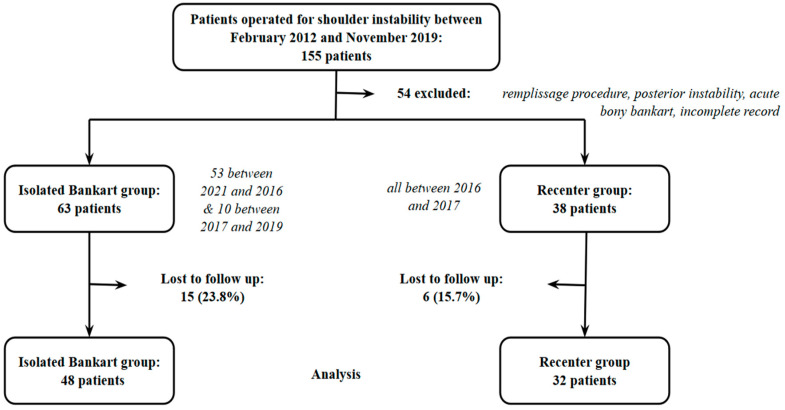
Participant flowchart.

Participants eligible for the “control” group consisted of 63 patients who underwent ABR in the main participating center. Fifty-three patients were operated on between February 2012 and March 2016, prior to the “Recenter” group’s period, and ten were operated on between November 2017 and November 2019. Fifteen of them were lost to follow-up, leaving forty-two patients included in this group (Figure 2).

### 2.5. Indication for the Technique

The Indication for both techniques was any anterior shoulder instability including recurrent dislocations or subluxations with an Instability Severity Index Score (ISIS) [19] inferior to 6 at the time of the study and subcritical glenoid bone loss (<20%). Bone defects were assessed on plain radiographs (3 rotations and Lamy views). If a deep or medial Hill–Sachs lesion or a bony Bankart was detected, a CT scan was performed to evaluate the GBL according to the best fit circle method [20].

### 2.6. ABR Technique

The procedures were performed by eight trained senior surgeons proficient in standard shoulder arthroscopy techniques. A standard arthroscopic capsulolabral reinsertion was made after glenoid preparation with at least two knotless anchors.

### 2.7. Recenter Surgical Technique

All procedures were performed by the same surgeons under general anesthesia and an interscalene block, using arthroscopic techniques.

The patient was positioned in either the beach chair position or the lateral position with distal traction, depending on the surgeon’s preference.

#### 2.7.1. Step 1: Diagnostic Arthroscopy and Anterior Preparation

A posterior (P) portal is established in line with the joint to examine the intra-articular structures. The starting point is slightly more medial than a normal portal to allow the later placement of the arthroscopic specific guide. Injuries of the soft tissue and bone loss (on the humeral side and/or on the glenoid side) as well as concomitant lesions are assessed. A second antero-medial (AM) portal is established with a needle through the rotator interval to facilitate the access of the antero-inferior part of the glenoid. It is important to thoroughly debride the rotator interval with an arthroscopic radiofrequency ablation (RFA) device. This wide opening will help the passage of the Recenter.

#### 2.7.2. Step 2: Glenoid Preparation

With the camera maintained in the P portal, the anterior glenoid rim is prepared, with an RFA device and freshened with a motorized rasp (Power Rasp Arthrex, Naples, FL, USA) through the AM portal, between 3 and 5 o’clock. The labrum must be fully mobilized from the glenoid rim until the fibers of the subscapularis are visualized to create enough space for the Recenter. Then, the Bankart repair is prepared by drilling two or three holes for the knotless anchors (Push lock 2.9, Arthrex, Naples, FL, USA).

#### 2.7.3. Step 3: Tunnel Preparation

**Figure 3:**
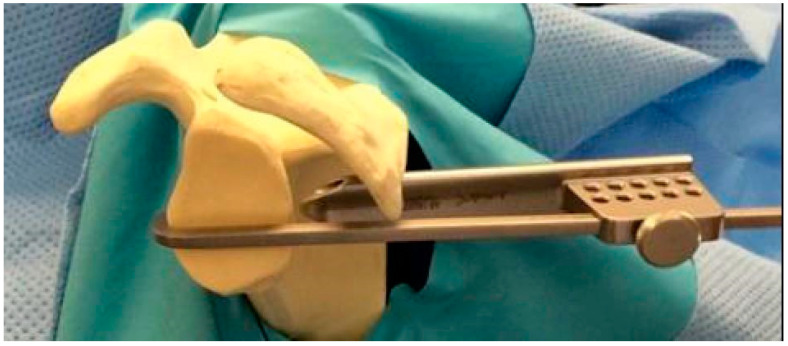
Specific arthroscopic guide.

A third antero-lateral (AL) viewing portal is created. The arthroscopic guide is then introduced posteriorly, and the hook is positioned at 4 o’clock, parallel to the glenoid surface (Figure 3). The hook allows parallel drilling with an exit hole 7 mm medial to the glenoid to prevent the lateral overhang of the implant. Two guide pins are drilled through the guide. Once their position is judged satisfactory (3 o’clock for the superior pin and 5 o’clock for the inferior one), two 4 mm tunnels are drilled using a cannulated drill. The two drills are left in place and the guide pins are removed.

#### 2.7.4. Step 4: Implant Positioning

**Figure 4:**
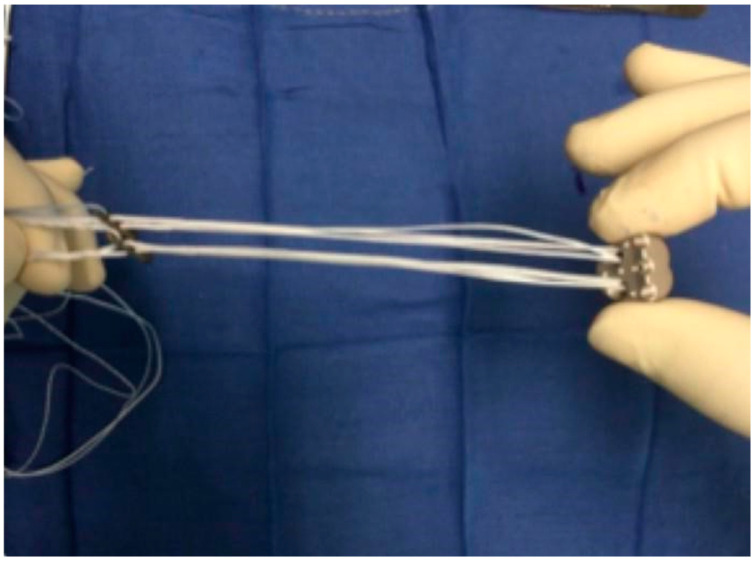
Recenter mounted with the two knotless TightRopes.

The arthroscope is maintained in the AL portal and two suture passers (Nitinol, Arthrex, Naples, FL, USA) are shuttled through the cannulated drills, exiting via the AM portal. Two cortical buttons (CBs) (Knotless TightRope, Arthrex, Naples, FL, USA) are passed through the Recenter (Figure 4) and are shuttled through the glenoid using the superior and inferior cannulated drills. The sutures are pulled posteriorly while the Recenter is guided inside the joint through the rotator interval.

Once the position of the Recenter is deemed satisfactory, the implant is secured by tightening with the two CBs posteriorly using a suture tensioner.

#### 2.7.5. Step 5: Bankart Repair

**Figure 5:**
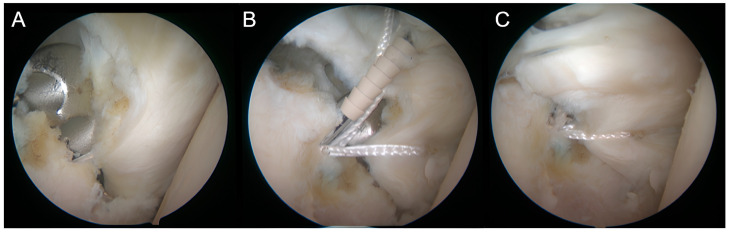
Recenter in position before and after Bankart repair. A *Recenter* implanted, B Anchor fixing the labrum, C Final aspect.

A standard Bankart repair is then performed with knotless anchors (Push lock 2.9, Arthrex, Naples, FL, USA) in order to place the implant in an extra-articular position (Figure 5).

### 2.8. Postoperative Management

After surgery, in both groups, shoulders were immobilized in a shoulder sling with the arm in internal rotation for 1 month. Physiotherapy began at one month postoperatively with active assisted rehabilitation, then progressed to active motion with shoulder strengthening starting at 3 months postoperatively. Return to sporting activity was allowed 5–6 months after surgery. In the “Recenter” group, the radiographic confirmation of implant stability occurred at one month.

### 2.9. Outcome Measures

The primary outcome measure was the dislocation recurrence rate. Secondary outcome measures included the following: the auto Walch–Duplay score (WD) [21] which is a questionnaire filled by the patient based on the Walch–Duplay score, the Simple Shoulder Test (SST), the auto Rowe score, the rate of reintervention, complications, return to sports, satisfaction, pain, the range of motion, and the presence or absence of subjective subluxation and apprehension (the patient stating is “I fear my shoulder can dislocate” or “I feel my shoulder can dislocate”).

For the “Recenter” group, a subgroup analysis was performed to assess the orientation of the device. All patients underwent AP and sagittal view radiographs and a postoperative 3D CT scan at one month postoperatively. The correct positioning of the Recenter was evaluated using the CT scan analysis method described for the Latarjet by Kraus et al. [22] that has shown good intra-observer reproducibility and inter-observer reproducibility. The assessment was performed using an open access version of OsiriX TM (OsiriX Switzerland, image processing software, version 3.8). The orientation of the Recenter was determined by measuring the alpha (α) angle on axial cuts using the CB tunnel axis [23]. The surgical times were also measured in this group.

### 2.10. Data Collection

Patients from each group were contacted after a minimum follow-up of 2 years. A questionnaire was completed by the patients themselves. Patients who did not respond were contacted by phone and email three times before being considered lost to follow-up. This same questionnaire was submitted in the “Recenter” group. Preoperative data were collected retrospectively from the medical records.

### 2.11. Statistical Analysis

Qualitative variables, described by the number of events and their percentage, were compared using either the Pearson’s Chi2 test or the Fisher exact test, depending on the group sizes. Quantitative data were described by means and standard deviations and were compared by the appropriate statistical tests between Student’s *t* test or the Mann–Whitney test. Test selection was based on data characteristics: parametric tests for normally distributed variables (the Shapiro–Wilk test) and non-parametric tests otherwise.

A *p* value < 0.05 was considered statistically significant. All statistical analyses were carried out using the R software (version 4.2).

## 3. Results

### 3.1. Demographics

A total of 32 patients were evaluated in the “Recenter” group, with an average follow-up of 64 months (minimum 26 and maximum 78), compared to 48 patients in the control group, with an average follow-up of 77 months (minimum 26 and maximum 114). Despite the significant difference in follow-up duration between the two groups, no other differences were observed, including in terms of age, gender, the dominant side of the operated shoulder, the level of sports activity, or the ISIS. The characteristics of the study populations are summarized in Table 1.

### 3.2. Recurrence Rate

At a mean follow-up of 5.4 years, three patients (9.4%) reported at least one postoperative dislocation in the “Recenter” group versus eight patients (16.7%) in the control group at a mean follow-up of 6.4 years (*p* > 0.05). Five patients out of forty-eight went under a revision surgery in the control group versus none in the “Recenter” group (*p* > 0.05).

### 3.3. Secondary Outcomes

The average operative time was 90.9 ± 18.9 min (60–130). Operative times decreased as the number of surgeries already performed increased. There were no implant-related intra-operative or postoperative complications, and there were no vascular or nerve injuries in either group. During the initial follow-up, no glenohumeral osteoarthritis was detected. There were no statistical differences in secondary outcomes. The results are shown in Table 2.

### 3.4. Early Radiologic Results

**Figure 6:**
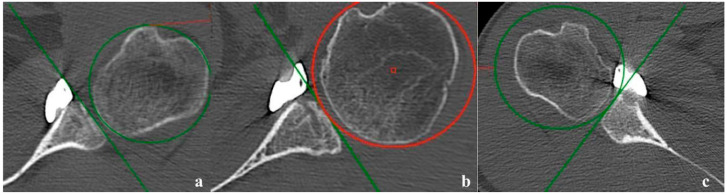
Position of the Recenter (**a**) flush, (**b**) congruent and (**c**) lateral.

**Figure 7:**
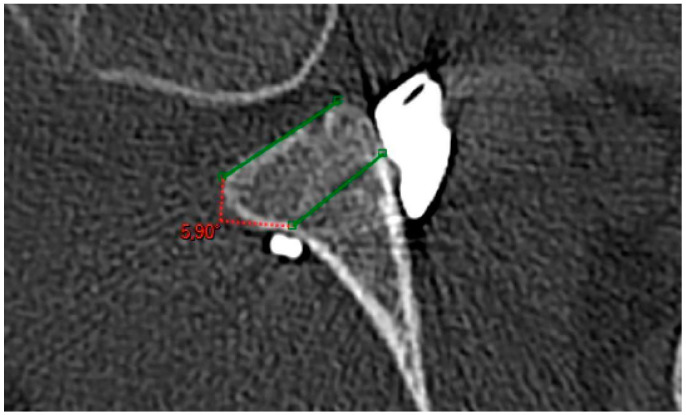
α angle of the Recenter.

One-month postoperative 3D CT scans were performed in all patients. The Recenter implant was in a medial position (>1 mm medially to the glenoid axis) in 10 (31.3%) cases, flush (less than 1 mm to the glenoid axis) in 16 (50%) cases, congruent (>1 mm laterally to the glenoid axis) in 5 (15.7%) cases and lateral (laterally to a circle with the radius the shortest distance between the center of the humeral head and the glenoid) in 1 (3%) case (Figure 6). More precisely, the implant was at a mean distance of 1.6 ± 1.8 mm (0; 6.5) of the tangential line. When assessing sagittal plane alignment, the center of the Recenter was placed between three and four o’clock in 31 cases (97%), and up to three o’clock in 1 (3%) case. The mean α angle was 5.3 ± 3.9° (0; 11.6) (Figure 7).

## 4. Discussion

The results of this unprecedented technique gave good postoperative stability with no evidence of augment migration or failure. At the last follow-up, none of the clinical scores were significantly better in the “Recenter” group, but we think there could be a small trend towards an improvement with the Recenter. In the case of the patient whose metal bone block was placed laterally, the result was surprisingly good: there was no recurrence or re-operation; he was pain-free and not limited in his sport of weight training. However, he still has limited second external rotation and internal rotation. Outcomes in the control group were similar to other series in the literature, thus making the comparison worthy [4,5,19,24,25,26,27].

Our hypothesis is that a block that reduces the antero-inferior translation of the head would allow the better healing of the soft tissues of the Bankart repair, thus providing better functional outcomes and a lower recurrent instability rate. There are other techniques described to improve ABR with a block supporting the capsulolabral repair and they seem to have good results, succeeding to lower the dislocation rate. Iizawa [15] compared his Bankart technique augmented by reconstruction, either with an iliac autograft or a hydroxyapatite substitute, with the Bankart technique alone in patients with a glenoid defect of more than 20%. He found a significant improvement over 2 years in the dislocation rate (2.9% vs. 48.5%) and Rowe score (95 vs. 70 out of 100). Taverna [28] studied ABR with an underlying reconstruction of the glenoid by an iliac crest allograft in a population of 26 patients, unstable for less than 3 years, with less than five dislocations and either unipolar lesions of the glenoid greater than 15% or bipolar lesions with more than 10% glenoid bone loss (GBL). These inclusion criteria were chosen because this is a population at risk of recurrence during isolated ABR but with tissue quality relatively spared by the number of episodes and the duration of instability. The results are good at 2.5 years with a zero-recurrence rate and a Rowe score of 96. The graft was fixed with two endobuttons with the same kind of arthroscopic posterior guide as for the Recenter. The positioning of the graft was flush in all cases and high in only one case.

Avramidis [29] and Giannakos [30] used the iliac crest in case of bone loss or to deal with a previous failure with a very low rate of recurrence, but Giannakos had to remove his screws in 33% of his patients. In comparison, Avramidis had no hardware removal using the endobutton system. Finally, Boileau [31] had good results treating Latarjet failures complicated with >20% glenoid bone loss with the same type of arthroscopic Eden Hybinette. His iliac autografts were placed extra-articular by reinserting the remaining capsulolabral complex above them in the same manner as in the other studies. The results of these bone block procedures are detailed in Table 3.

Adding the implantation of the Recenter to the surgical technique adds time and requires a few more skills. However, the placement of the implant, performed by several surgeons, was good in most cases. The use of a posterior guide for precise endobutton placement and shuttling the block through the rotator interval is an already known and reproducible technique. It is also described in other surgeries such as the arthroscopic Eden Hybinette and the arthroscopic Latarjet [28,29,31]. We believe that using the posterior guide with the endobutton system is less difficult than placing a screw through a subscapularis split. The risk of axillary nerve injury is also lower when passing through the rotator interval.

The initial choice was to fix the recenter with two screws, but to facilitate its installation, avoid screw removal and guarantee reproducible positioning, the TightRope system was chosen.

Unlike previous studies, the desired effect is not a permanent increase in the anterior rim of the glenoid, whose congruence is improved by wrapping the labrum above. The concept is the opposite: it is tissue repair supported by a buttress. As with a usual Bankart procedure, this involves repairing the main anterior tissue break on humeral translation. The theorical improvement is to promote the robustness of the capsulolabral scar by decreasing the stress pulled by the antero-inferior translation of the head when healing. We bet that by using the Recenter for moderate glenoid bone loss, the implant would remain embedded in the fibrous tissue of the adjacent capsulolabral healing after a few months. Because its role is meant to be temporary, the implant does not have a surface dedicated to bony integration on the scapular neck. In case of a revision, it would be easy to remove. The main advantage of a standard metal block over an allograft is its cost. It can be easily available. Advantages over an allograft is that it does not need to be shaped nor harvested as it is ready to use.

### Limitation

There are several limitations of this study. The main limitation is the retrospective reconstitution of the two groups. The recenter group included all patients who received this implant at all centers. In the main center, in which the promoter of the technique worked, patients systematically received the implant when a Bankart indication was established during the testing phase. The other centers were private clinics where some surgeons trained at the main center wished to use the implant. Two-thirds of the “Recenter” patients underwent surgery at the main center. The promoter of the technique died before the end of his trials, and the implant was no longer available. The control group was set up retrospectively. For practical reasons of accessibility to records, it was decided to include only patients from the main center, although this introduces a bias. Patients who had a simple Bankart were therefore sought before and after the trial phase. The first patient included in the “control” group corresponds to the first patient known to the current informatic system with an available medical record. Shortly after the trial period, the team changed the paradigm to follow the mainstream in our country: we now favor open Latarjet because of its low recurrence rate [9]. Currently we indicate ABR only when ISIS is less than or equal to 1. This explains the smaller number of patients in the control group after the test phase. Thus, there was a statistical difference in the follow-up with shorter follow-up for the Recenter. This difference in favor of the augmented group is a confounding factor increasing the risk, leading us to conclude that the hypothesis of a lower recurrence rate in this group is true. However, we found no difference between groups, failing to find true interest in this new technique. But still, we could have ignored significant worse outcomes with the augmented group.

Concerning the indications for ABR at the time of this study, a systematic CT scan was not routinely performed to quantify bony lesions. Plain radiographs were supplemented by a CT scan if there was concern about problematic humeral or glenoid bone loss or the combination of the two. So, we may have underestimated bone loss and lacked precision to quantify the overall glenoid bone loss in this series, even if a positive glenoid bony lesion on the ISIS has been associated with a lesion greater than 15% of the glenoid [32]. There was no calculation of the glenoid track because we relied mainly on the ISIS; this study is therefore difficult to compare with recent studies.

Bohem [33] published a series of 14 patients operated on for an arthroscopic Eden Hybinette with the repair of a Bankart lesion around the graft and not above it. She found that on a CT scan, the area of the articular surface, grossly increased by the graft, decreased until it reached the size of the contralateral glenoid articular surface at the last follow-up. This demonstrates the remodeling power of living bones according to Wolf’s law. The lack of remodeling with the Recenter could lead to an excessively strong block causing osteoarthritis over time with the aging of the adjacent tissue. On the other hand, the lack of bony fixation could lead to the mobilization of the implant, creating an exhaustion of the armed labral repair and recurrent instability. Another limitation of this study is the absence of radiographic follow-up. How do implants behave over time? They could be responsible for osteoarthritis or osteolysis at the neck of the scapula. They could migrate over time, even in the joint. Instability arthropathy (AI) is a subject that is still obscure to this day. The specific responsibility of each technique in the progression of AI is poorly understood. Rather, it is the risk factors linked to the patient and the history of instability that are identified [34]: multiple dislocations, no surgical stabilization, over 25 years old at the time of the first episode, alcoholism, and occurrence due to high energy trauma. Concerning the techniques themselves, it seems that the risk situations for AI are the failure of stabilization and the conflict with the humeral head (by a block, an anchor or a screw) [35]. In our series, there was only one lateralized implant postoperatively.

Several studies have demonstrated that the soft tissue procedure recurrence rate may increase over time [4,5]; it seems to be the same case here with the metal glenoid augmentation. Today, we would not try this implant again for two reasons. First, the idea of a temporary useful block to address permanent instability is daring and arguable, and the examples of glenoid bone grafts discussed above already show appealing results. Second, we have already taken a shift towards Latarjet which is currently the gold standard in our country. Third, the results of this study show no significant improvement in outcomes with the Recenter implant compared to traditional ABR, making it an unnecessary augmentation for this indication. In our opinion, the technique of ABR augmentation with Recenter will not fill the gap between the indication of standard ABR and the Latarjet procedure for patients suffering several dislocations with no or subcritical bone defects.

The small sample size is a true limitation in this study, which may have contributed to the insufficient statistical power to detect differences between the groups. As a result, the non-significant *p*-values should be interpreted with caution, reflecting the lack of evidence to support differences under the current study conditions rather than confirming the absence of differences. In addition, another limitation of this study is the theoretical risk of inflated type I error rates due to multiple univariate analyses, though this risk is minimized as none of the *p*-values reached the significance threshold.

## 5. Conclusions

ABR safely restores shoulder stability in selected patients with subcritical glenoid bone deficiency. However, the addition of the Recenter metal implant did not improve outcomes compared to traditional Bankart repair. The technique, which has since been abandoned, adds presumed significant surgical time and technical challenges without offering clear benefits. We do not recommend using it to extend ABR indications towards greater bone deficiency, as existing alternatives such as the Latarjet procedure remain more reliable and effective in addressing recurrent instability.

## Figures and Tables

**Table 1:** Demographics of patients.

	Recenter Group n = 32	Control Group n = 48	*p*-Value
Age in year	32 ± 3.6	31 ± 2.8	>0.05
Female Gender	10 (31%)	21 (44%)	>0.05
Dominant Side Operated on	22 (71%)	25 (52%)	>0.05
Sport Practice			>0.05
Competition	6 (19%)	14 (29%)	
Recreational	23 (72%)	33 (69%)	
None	3 (9%)	1 (2%)	
Contact or Combat Sports	13 (41%)	28 (58%)	>0.05
ISIS	2.1 ± 0.7	1.8 ± 0.5	>0.05
Follow-Up in Year	5.4 ± 0.3	6.4 ± 0.5	<0.0001
Loss to Follow-Up	6 out of 38 (16%)	15 out of 63 (29%)	>0.05

**Table 2:** Functional outcomes.

Scoring System	*Recenter* Group n = 32	Control Group n = 48	*p*-Value
Recurrence rate, %	3 (9.4%)	8 (16.7%)	>0.05
Revision, %	0 (0%)	5 (10.4%)	>0.05
Subluxation, %	2 (6.3%)	2 (4.2%)	>0.05
Apprehension, %	9 (28.1%)	14 (29.2%)	>0.05
Walch–Duplay score	70.2 ± 8.2	64.2 ± 8	>0.05
Rowe score	75.3 ± 9	69.2 ± 7.8	>0.05
SST, %	84.6 ± 6	81.5 ± 5.5	>0.05
Satisfaction, %	28 (87.5%)	37 (77.1%)	>0.05
Numeric pain rating scale, pts	1.4 ± 0.6	1.4 ± 0.6	>0.05
Pain in daily living, %	2 (6.3%)	8 (16.7%)	>0.05
Return to same level of sport, %	18 (56.3%)	23 (47.9%)	>0.05
Return to same sport, %	27 (84.4%)	42 (87.5%)	>0.05
Abduction < 150°, degree	12.5 ± 11.6	6.3 ± 6.9	>0.05
Loss > 30% of ER2, degree	12.5 ± 11.6	14.6 ± 10.1	>0.05
Loss > 10 cm of IR, degree	28.1 ± 15.8	31.3 ± 13.3	>0.05

ER2: 2nd external rotation, IR: internal rotation.

**Table 3:** Studies showing ABR completed with bone block procedures.

	IIzawa [15]	Taverna [25]	Avramidis [26]	Giannakos [27]	Boileau [28]	«Recenter» Group
Year	2019	2018	2020	2017	2019	2024
Size	28 vs. 7	26	28	12	7	27
Follow-up in year	2.5	2.5	3.5	2.4	1.5	3
Indication	GBL > 20%	ISIS > 3+ GBL > 10% +Nb Disl < 5	GBL > 8% 1st Int or Revision	revision	Latarjet failure + GBL > 20%	recurrent dislocation, ISIS < 6, GBL < 20%, 1st Int surgery
Type of graft	iliac crest vs. substitute	allograft	iliac crest	iliac crest	iliac crest	metal block
Fixation	screws vs. anchors	endobutton	endobutton	screws	endobutton	endobutton
Recurrence %	3	0	0	0 (12% SBlux)	14	4
Rowe score	95	96	90	70	86	83
>50% Ostéolysis %	14 vs. 0	4	0	8	no data	-
Malunion %	0 vs. 25	4	0	33	0	-
Comments	ABR alone: 50% recurrence	100% of grafts flush	94% of grafts flush	screw removal 33%	100% of grafts flush	47% of grafts flush (1 mm margin)

GBL: glenoid bone loss; Disl: dislocation; SBlux: subluxation; 1st Int: first intention.

## Data Availability

The original contributions presented in the study are included in the article, further inquiries can be directed to the corresponding authors.
